# Crystal structure of 4,10-dimeth­oxy-13-methyl-6*H*,12*H*-6,12-epimino­dibenzo[*b*,*f*][1,5]dioxocine

**DOI:** 10.1107/S2056989017002328

**Published:** 2017-02-21

**Authors:** Katerina V. Kasyanova, Vladimir N. Kokozay, Elena A. Buvaylo, Olga Yu. Vassilyeva, Brian W. Skelton

**Affiliations:** a Department of Chemistry, Taras Shevchenko National University of Kyiv, 64/13 Volodymyrska Street, Kyiv 01601, Ukraine; b School of Molecular Sciences, M310, University of Western Australia, Perth, WA 6009, Australia

**Keywords:** crystal structure, *o*-vanillin, methyl­amine hydro­chloride, Schiff base, self-condensation, [1,5]dioxocin ring, twisted-boat conformation

## Abstract

The title compound, C_17_H_17_NO_4_, lacks crystallographic symmetry with one mol­ecule per asymmetric unit. The mol­ecule exists in a folded butterfly-like conformation; the benzene rings form a dihedral angle of 84.72 (7)°. The central eight-membered imino-bridged dioxocin ring adopts a twisted-boat conformation. In the crystal, inversion-related mol­ecules are linked by pairs of weak C—H⋯O hydrogen bonds, forming double-stranded chains parallel to the *a* axis.

## Abstract 2

The Schiff base mol­ecule is transformed into a substituted dibenzo­imino­[1,5]dioxocin compound featuring a folded butterfly-like conformation with a dihedral angle of 84.72 (7)° between the benzene rings.

## Chemical context   

Tröger’s base and its structural analogues are characterized by two flat, usually aromatic and identical, pincers inter­locked in an almost perpendicular fashion (Dolenský *et al.*, 2012 ▸). Both the chirality and the conformational rigidity of their central diazo­cine, dioxocin or di­thio­cin skeletons are the reasons why these cleft-shaped mol­ecules have been of inter­est in mol­ec­ular recognition (Hardouin-Lerouge *et al.*, 2011 ▸), as chiral solvating agents (Wilen *et al.*, 1991 ▸), and in the field of asymmetric synthesis (Minder *et al.*, 1995 ▸).
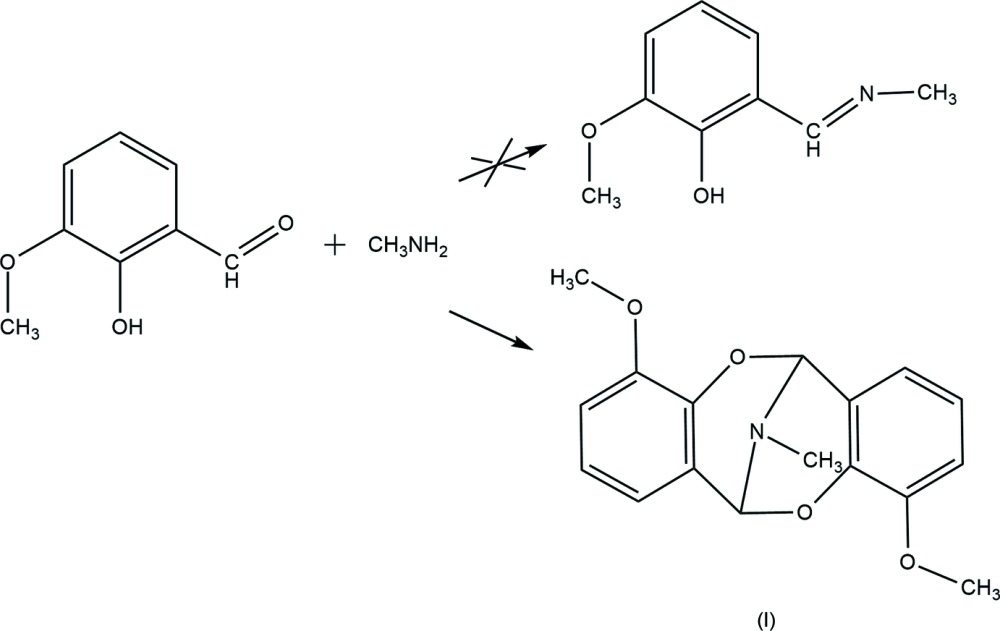


Over the last few years, we have been exploring the chemistry of transition metal complexes of Schiff base ligands with the aim of preparing heterometallic polynuclear compounds with diverse potential advantages (Chygorin *et al.*, 2012 ▸; Nesterova *et al.*, 2013 ▸). The Schiff base ligand 2-meth­oxy-6-imino­methyl­phenol (H*L*) with various connectivity modes has been successfully used as a multidentate linker between several metal centres by our group and others (Meally *et al.*, 2010 ▸; Sydoruk *et al.*, 2013 ▸). The H*L* ligand is usually obtained by the standard method of condensation of the substituted salicyl­aldehyde with an aqueous solution of methyl­amine in methanol (Meally *et al.*, 2010 ▸). In the present work, we used a mixture of 2-hy­droxy-3-meth­oxy-benzaldehyde and methyl­amine hydro­chloride to react with a zinc salt in an attempt to synthesize a Zn complex with the H*L* ligand (see Scheme). The resulting Schiff base apparently underwent self-condensation to form the substituted dibenzo­imino­[1,5]dioxocin, 4,10-dimeth­oxy-13-methyl-6*H*,12*H*-6,12-epimino­dibenzo[*b*,*f*][1,5]dioxocine, (I), the crystal structure of which is presented here. A close analogue of the title compound was reported to result from 2-(*N*-methyl­imino­meth­yl)phenol, a liquid product of a similar condensation of salicyl­aldehyde and methyl­amine, after a few months storage in mild conditions (Filarowski *et al.*, 1998 ▸). A tentative mechanism for the formation of the [1,5]imino­dioxocin ring in the reaction between an aromatic aldehyde and a primary amine was given by Mandal *et al.* (2006 ▸).

## Structural commentary   

**Figure 1:**
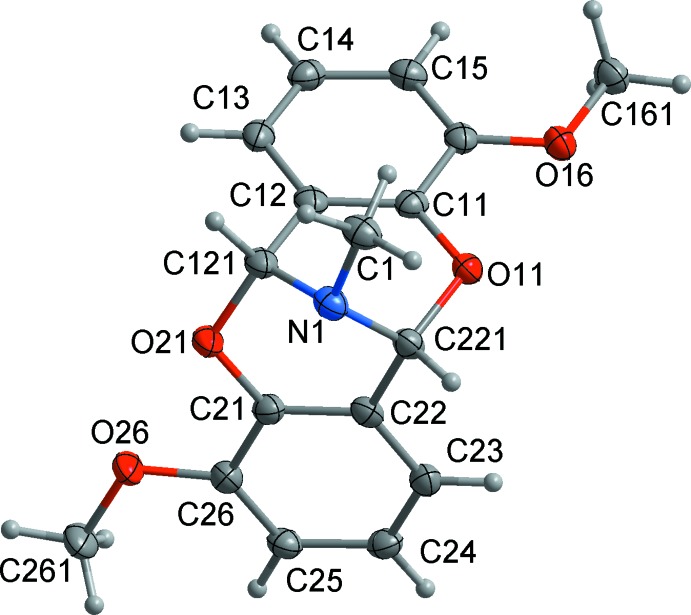
The mol­ecular structure of the title compound, showing the atom-numbering scheme. Non-H atoms are shown with displacement ellipsoids at the 50% probability level.

The title compound is composed of four fused rings including two benzene (C11–C16 and C21–C26) and two six-membered heterocyclic rings (O11/C11/C12/C121/N1/C221 and O21/C21/C22/C221/N1/C121) (Fig. 1 ▸). The organic molecule has two chiral centres and lacks crystallographic symmetry; the crystal is racemic. The molecule exists in a folded butterfly-like conformation with a dihedral (folding) angle between the two benzene rings of 84.72 (7)°. The eight-membered imino-bridged dioxocin ring adopts a twisted-boat conformation, as judged from the eight torsion angles observed within this ring (τ_1_–τ_8_) (Mandal *et al.*, 2006 ▸). The bond lengths and angles are unexceptional and are closely related to those of *N*-methyl-2,6,-dioxa-9-aza-(*c*,*g*)dibenzo(3.3.1)nonane (CSD refcode UCERIE; Filarowski *et al.*, 1998 ▸); the dihedral angle of 84.72 (7)° is larger than that in the unsubstituted imino­dioxocin mol­ecule (80.95°).

## Supra­molecular features   

**Figure 2:**
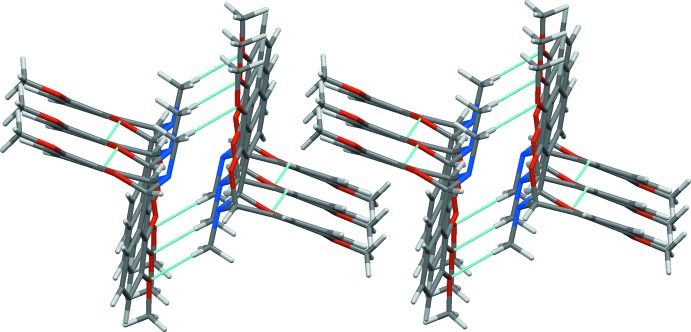
Crystal packing of (I), showing the parallel arrangement of double-stranded hydrogen-bonded chains of the dibenzo­imino­[1,5]dioxocin mol­ecules along the *a*-axis direction. Inter­molecular hydrogen bonds are shown as blue dashed lines.

In the crystal, double-stranded chains of inversion-related mol­ecules linked by pairs of weak C–H⋯O hydrogen bonds (Table 1 ▸) propagate in the *a*-axis direction (Fig. 2 ▸). Adjacent hydrogen-bonded chains are arranged in a parallel fashion to ensure efficient crystal packing of the clefts. Surprisingly, neither π–π stacking [the shortest centroid–centroid distance (offset) = 3.96 Å] nor C—H⋯π inter­actions (the shortest H⋯centroid distance = 3.34 Å) play a significant role in formation of the crystal structure of (I).

## Database survey   

More than 1000 crystal structures of mol­ecules featuring eight-membered heterocine rings with two oxygen atoms in a 1,2-, 1,3-, 1,4- and 1,5-relationship, both uncondensed and fused to five-, six-, and seven-membered carbocycles or heterocycles, are found in the Cambridge Structural Database (CSD Version 5.37 plus one update; Groom *et al.*, 2016 ▸) with bridged dioxocines constituting the majority of the compounds reported. Of theses, only five mol­ecules contain the same central imino-bridged [1,5]dioxocin core as in compound (I) (refcodes GAQNUJ, QAYTIU, TECMAP, UCERIE, XESBON). Clearly, substituents on the aromatic rings and on the bridging imino N atom in the five compounds determine the differences in their folding angles, which fall in the range 78.49–96.84°. However, no obvious correlation between the nature/size/position of the substituents and widening of the folding angle can be established due to the small number of compounds involved. While an example of [1,5]imino­dioxocin bridgehead N-atom coordination to a metal atom (copper) has been reported (refcode XESBON; Mandal *et al.*, 2006 ▸), the Zn atom did not demonstrate the ability to coordinate the ligand (I) in the present study.

## Synthesis and crystallization   

2-Hy­droxy-3-meth­oxy-benzaldehyde (0.23 g, 1.5 mmol) and methyl­amine hydro­chloride (0.10 g, 1.5 mmol) were added to methanol (5 ml) and stirred magnetically for 10 min. Zn(CH_3_COO)_2_·2H_2_O (0.11 g, 0.5 mmol) dissolved in 5 ml di­methyl­formamide was added to the yellow solution of the Schiff base formed *in situ*, and the resulting deep-yellow solution was stirred at room temperature for an hour. The addition of N(Et)_3_ (1 ml) produced a light precipitate which was filtered off. The solution, which was kept cold (283–285 K), changed colour from yellow to brown. It was diluted twice with methanol (4 ml) since it was thickening. Brown plate-like crystals of the title compound formed over two months after successive addition of Pr^*i*^OH (4 ml) in two portions. They were collected by filter-suction, washed with dry Pr^*i*^OH and finally dried in air (yield: 23%). Analysis calculated for C_17_H_17_NO_4_ (299.31): C, 68.21; H, 5.72; N, 4.68%. Found: C, 68.55; H, 5.49; N, 4.87%. ^1^H NMR (400 MHz, DMSO-*d*_6_, *s*, singlet; *m*, multiplet): δ (ppm) 6.89–6.79, *m* (6H, benzene rings); 5.69, *s* (2H, dioxocin ring); 3.71, *s* (6H, OCH_3_); 2.51, *s* (3H, NCH_3_). The IR spectrum of powdered (I) in the range 4000–400 cm^−1^ shows all characteristic functional groups peaks: ν(CH) due to aromatic =C—H and alkyl –C—H stretching above and below 3000, respectively, the aromatic rings vibrations in the 1600–1400 region, ν(CO) and ν(CN) at 1300–1000 and aromatic CH bending in the 900–600 cm^−1^ region (see Supporting information).

## Refinement   

Crystal data, data collection and structure refinement details are summarized in Table 2 ▸. All hydrogen atoms bound to carbon were included in calculated positions and refined using a riding model with isotropic displacement parameters based on those of the parent atom (C—H = 0.95 Å, *U*_iso_(H) = 1.2*U*_eq_C for CH, C—H = 0.98 Å, *U*_iso_(H) = 1.5*U*_eq_C for CH_3_). Anisotropic displacement parameters were employed for the non-hydrogen atoms.

## Supplementary Material

Crystal structure: contains datablock(s) I, global. DOI: 10.1107/S2056989017002328/hg5482sup1.cif

Structure factors: contains datablock(s) I. DOI: 10.1107/S2056989017002328/hg5482Isup2.hkl

IR spectrum in KBr. DOI: 10.1107/S2056989017002328/hg5482sup3.pdf

Supporting information file. DOI: 10.1107/S2056989017002328/hg5482Isup4.cml

CCDC reference: 1532218

Additional supporting information:  crystallographic information; 3D view; checkCIF report

## Figures and Tables

**Table 1:** Hydrogen-bond geometry (Å, °)

*D*—H⋯*A*	*D*—H	H⋯*A*	*D*⋯*A*	*D*—H⋯*A*
C13—H13⋯O11^i^	0.95	2.69	3.5616 (19)	152
C1—H1*B*⋯O26^ii^	0.98	2.53	3.508 (2)	176

Symmetry codes: (i) 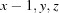; (ii) 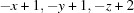.

**Table 2:** Experimental details

Crystal data	
Chemical formula	C_17_H_17_NO_4_
*M*_r_	299.31
Crystal system, space group	Triclinic, *P* 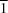
Temperature (K)	100
*a*, *b*, *c* (Å)	6.9956 (5), 8.8589 (6), 12.0938 (9)
α, β, γ (°)	93.980 (6), 106.603 (7), 102.133 (6)
*V* (Å^3^)	695.46 (9)
*Z*	2
Radiation type	Cu *K*α
μ (mm^−1^)	0.84
Crystal size (mm)	0.18 × 0.06 × 0.04

Data collection	
Diffractometer	Oxford Diffraction Gemini
Absorption correction	Multi-scan (*CrysAlis PRO*; Rigaku OD, 2015 ▸)
*T*_min_, *T*_max_	0.818, 1
No. of measured, independent and observed [*I* > 2σ(*I*)] reflections	5235, 2456, 2147
*R*_int_	0.027
(sin θ/λ)_max_ (Å^−1^)	0.598

Refinement	
*R*[*F* ^2^ > 2σ(*F* ^2^)], *wR*(*F* ^2^), *S*	0.041, 0.121, 1.07
No. of reflections	2456
No. of parameters	202
H-atom treatment	H-atom parameters constrained
Δρ_max_, Δρ_min_ (e Å^−3^)	0.22, −0.25

Computer programs: *CrysAlis PRO* (Rigaku OD, 2015 ▸), *SHELXT* (Sheldrick, 2015*a* ▸), *SHELXL2014* (Sheldrick, 2015*b* ▸), *DIAMOND* (Brandenburg, 1999 ▸), *Mercury* (Macrae *et al.*, 2006 ▸) and *WinGX* (Farrugia, 1999 ▸).

## supplementary crystallographic information

### Crystal data

**Table:**

C_17_H_17_NO_4_	*Z* = 2
*M**_r_* = 299.31	*F*(000) = 316
Triclinic, *P*1	*D*_x_ = 1.429 Mg m^−^^3^
Hall symbol: -P 1	Cu *K*α radiation, λ = 1.54184 Å
*a* = 6.9956 (5) Å	Cell parameters from 2735 reflections
*b* = 8.8589 (6) Å	θ = 3.9–67.1°
*c* = 12.0938 (9) Å	µ = 0.84 mm^−^^1^
α = 93.980 (6)°	*T* = 100 K
β = 106.603 (7)°	Plate, brown
γ = 102.133 (6)°	0.18 × 0.06 × 0.04 mm
*V* = 695.46 (9) Å^3^	

### Data collection

**Table:**

Oxford Diffraction Gemini diffractometer	2456 independent reflections
Radiation source: sealed X-ray tube	2147 reflections with *I* > 2σ(*I*)
Mirror monochromator	*R*_int_ = 0.027
Detector resolution: 10.4738 pixels mm^-1^	θ_max_ = 67.2°, θ_min_ = 3.9°
ω scans	*h* = −8→8
Absorption correction: multi-scan (CrysAlis PRO; Rigaku OD, 2015)	*k* = −9→10
*T*_min_ = 0.818, *T*_max_ = 1	*l* = −12→14
5235 measured reflections	

### Refinement

**Table:**

Refinement on *F*^2^	0 restraints
Least-squares matrix: full	Hydrogen site location: inferred from neighbouring sites
*R*[*F*^2^ > 2σ(*F*^2^)] = 0.041	H-atom parameters constrained
*wR*(*F*^2^) = 0.121	*w* = 1/[σ^2^(*F*_o_^2^) + (0.0737*P*)^2^ + 0.1321*P*] where *P* = (*F*_o_^2^ + 2*F*_c_^2^)/3
*S* = 1.07	(Δ/σ)_max_ < 0.001
2456 reflections	Δρ_max_ = 0.22 e Å^−^^3^
202 parameters	Δρ_min_ = −0.25 e Å^−^^3^

### Special details

**Table:**

Geometry. All esds (except the esd in the dihedral angle between two l.s. planes) are estimated using the full covariance matrix. The cell esds are taken into account individually in the estimation of esds in distances, angles and torsion angles; correlations between esds in cell parameters are only used when they are defined by crystal symmetry. An approximate (isotropic) treatment of cell esds is used for estimating esds involving l.s. planes.

### Fractional atomic coordinates and isotropic or equivalent isotropic displacement parameters (Å^2^)

**Table:**

	*x*	*y*	*z*	*U*_iso_*/*U*_eq_	
C1	0.8680 (3)	0.77719 (18)	0.96792 (13)	0.0272 (4)	
H1A	1.0173	0.8034	1.0043	0.041*	
H1B	0.802	0.8059	1.0249	0.041*	
H1C	0.8334	0.8345	0.9014	0.041*	
N1	0.7947 (2)	0.60859 (15)	0.92762 (11)	0.0239 (3)	
C11	0.6973 (2)	0.64283 (16)	0.69544 (13)	0.0208 (3)	
O11	0.89314 (15)	0.63442 (12)	0.75392 (9)	0.0216 (3)	
C12	0.5341 (2)	0.60187 (17)	0.74052 (13)	0.0220 (3)	
C121	0.5761 (2)	0.56411 (17)	0.86455 (13)	0.0234 (3)	
H121	0.5042	0.624	0.9056	0.028*	
C13	0.3363 (2)	0.60620 (17)	0.67238 (14)	0.0248 (3)	
H13	0.2232	0.5779	0.7016	0.03*	
C14	0.3059 (2)	0.65148 (18)	0.56296 (14)	0.0260 (4)	
H14	0.1707	0.6494	0.5159	0.031*	
C15	0.4714 (2)	0.70032 (17)	0.52068 (13)	0.0245 (3)	
H15	0.4492	0.7351	0.4465	0.029*	
C16	0.6678 (2)	0.69812 (17)	0.58676 (13)	0.0217 (3)	
O16	0.84270 (16)	0.74649 (12)	0.55663 (9)	0.0252 (3)	
C161	0.8176 (3)	0.80636 (19)	0.44818 (13)	0.0280 (4)	
H16A	0.7297	0.7244	0.3846	0.042*	
H16B	0.9522	0.8411	0.4367	0.042*	
H16C	0.7536	0.8946	0.4488	0.042*	
C21	0.6140 (2)	0.31265 (18)	0.82770 (12)	0.0214 (3)	
O21	0.50055 (16)	0.40035 (12)	0.86700 (9)	0.0242 (3)	
C22	0.8031 (2)	0.37774 (17)	0.81425 (12)	0.0215 (3)	
C221	0.8989 (2)	0.55005 (17)	0.85422 (13)	0.0218 (3)	
H221	1.0459	0.563	0.9011	0.026*	
C23	0.9039 (2)	0.28166 (18)	0.76738 (13)	0.0232 (3)	
H23	1.033	0.3251	0.7573	0.028*	
C24	0.8164 (2)	0.12416 (18)	0.73579 (13)	0.0241 (3)	
H24	0.8838	0.0603	0.7017	0.029*	
C25	0.6289 (2)	0.05727 (18)	0.75355 (13)	0.0235 (3)	
H25	0.5711	−0.0517	0.7332	0.028*	
C26	0.5290 (2)	0.15091 (18)	0.80075 (13)	0.0225 (3)	
O26	0.34536 (17)	0.10158 (12)	0.82185 (10)	0.0275 (3)	
C261	0.2625 (3)	−0.06296 (19)	0.80651 (16)	0.0326 (4)	
H26A	0.2318	−0.1056	0.7247	0.049*	
H26B	0.1359	−0.0843	0.8284	0.049*	
H26C	0.3628	−0.1118	0.8559	0.049*	

### Atomic displacement parameters (Å^2^)

**Table:**

	*U*^11^	*U*^22^	*U*^33^	*U*^12^	*U*^13^	*U*^23^
C1	0.0350 (9)	0.0211 (8)	0.0248 (8)	0.0057 (7)	0.0094 (7)	0.0021 (6)
N1	0.0294 (7)	0.0195 (7)	0.0237 (6)	0.0051 (5)	0.0100 (5)	0.0035 (5)
C11	0.0217 (7)	0.0147 (7)	0.0253 (7)	0.0047 (6)	0.0061 (6)	0.0013 (5)
O11	0.0214 (5)	0.0201 (5)	0.0240 (6)	0.0041 (4)	0.0077 (4)	0.0064 (4)
C12	0.0261 (8)	0.0138 (7)	0.0270 (8)	0.0045 (6)	0.0103 (6)	0.0020 (5)
C121	0.0282 (8)	0.0166 (7)	0.0290 (8)	0.0064 (6)	0.0134 (6)	0.0044 (6)
C13	0.0236 (8)	0.0165 (7)	0.0362 (8)	0.0042 (6)	0.0129 (6)	0.0023 (6)
C14	0.0239 (8)	0.0197 (8)	0.0331 (8)	0.0070 (6)	0.0057 (6)	0.0027 (6)
C15	0.0294 (8)	0.0199 (8)	0.0242 (7)	0.0073 (6)	0.0070 (6)	0.0043 (6)
C16	0.0250 (7)	0.0158 (7)	0.0253 (7)	0.0043 (6)	0.0099 (6)	0.0017 (6)
O16	0.0257 (6)	0.0272 (6)	0.0250 (6)	0.0060 (4)	0.0108 (4)	0.0084 (4)
C161	0.0327 (8)	0.0287 (9)	0.0257 (8)	0.0077 (7)	0.0125 (7)	0.0091 (6)
C21	0.0246 (7)	0.0202 (8)	0.0222 (7)	0.0080 (6)	0.0094 (6)	0.0046 (6)
O21	0.0282 (6)	0.0167 (6)	0.0330 (6)	0.0060 (4)	0.0169 (5)	0.0052 (4)
C22	0.0240 (7)	0.0201 (8)	0.0203 (7)	0.0054 (6)	0.0060 (6)	0.0063 (6)
C221	0.0239 (7)	0.0205 (8)	0.0218 (7)	0.0048 (6)	0.0075 (6)	0.0072 (6)
C23	0.0221 (7)	0.0261 (8)	0.0240 (7)	0.0073 (6)	0.0092 (6)	0.0075 (6)
C24	0.0281 (8)	0.0236 (8)	0.0251 (7)	0.0125 (6)	0.0101 (6)	0.0052 (6)
C25	0.0280 (8)	0.0176 (8)	0.0258 (7)	0.0063 (6)	0.0089 (6)	0.0046 (6)
C26	0.0227 (7)	0.0218 (8)	0.0249 (7)	0.0060 (6)	0.0091 (6)	0.0065 (6)
O26	0.0277 (6)	0.0177 (6)	0.0415 (6)	0.0035 (4)	0.0183 (5)	0.0060 (4)
C261	0.0319 (9)	0.0183 (8)	0.0505 (10)	0.0024 (7)	0.0196 (8)	0.0064 (7)

### Geometric parameters (Å, º)

**Table:**

C1—N1	1.4716 (19)	O16—C161	1.4271 (18)
C1—H1A	0.98	C161—H16A	0.98
C1—H1B	0.98	C161—H16B	0.98
C1—H1C	0.98	C161—H16C	0.98
N1—C221	1.433 (2)	C21—O21	1.3723 (18)
N1—C121	1.454 (2)	C21—C22	1.387 (2)
C11—O11	1.3707 (18)	C21—C26	1.407 (2)
C11—C12	1.394 (2)	C22—C23	1.399 (2)
C11—C16	1.410 (2)	C22—C221	1.515 (2)
O11—C221	1.4636 (17)	C221—H221	1
C12—C13	1.403 (2)	C23—C24	1.379 (2)
C12—C121	1.519 (2)	C23—H23	0.95
C121—O21	1.4413 (18)	C24—C25	1.403 (2)
C121—H121	1	C24—H24	0.95
C13—C14	1.381 (2)	C25—C26	1.381 (2)
C13—H13	0.95	C25—H25	0.95
C14—C15	1.395 (2)	C26—O26	1.3690 (19)
C14—H14	0.95	O26—C261	1.4286 (18)
C15—C16	1.382 (2)	C261—H26A	0.98
C15—H15	0.95	C261—H26B	0.98
C16—O16	1.3675 (19)	C261—H26C	0.98
			
N1—C1—H1A	109.5	H16A—C161—H16B	109.5
N1—C1—H1B	109.5	O16—C161—H16C	109.5
H1A—C1—H1B	109.5	H16A—C161—H16C	109.5
N1—C1—H1C	109.5	H16B—C161—H16C	109.5
H1A—C1—H1C	109.5	O21—C21—C22	122.61 (14)
H1B—C1—H1C	109.5	O21—C21—C26	116.68 (13)
C221—N1—C121	107.32 (12)	C22—C21—C26	120.72 (14)
C221—N1—C1	114.33 (12)	C21—O21—C121	111.48 (11)
C121—N1—C1	112.09 (12)	C21—C22—C23	119.15 (14)
O11—C11—C12	122.53 (13)	C21—C22—C221	119.33 (13)
O11—C11—C16	116.70 (13)	C23—C22—C221	121.48 (13)
C12—C11—C16	120.76 (13)	N1—C221—O11	111.99 (12)
C11—O11—C221	111.80 (11)	N1—C221—C22	109.13 (12)
C11—C12—C13	118.88 (14)	O11—C221—C22	110.50 (11)
C11—C12—C121	119.41 (13)	N1—C221—H221	108.4
C13—C12—C121	121.58 (13)	O11—C221—H221	108.4
O21—C121—N1	108.62 (12)	C22—C221—H221	108.4
O21—C121—C12	111.51 (12)	C24—C23—C22	120.23 (14)
N1—C121—C12	111.31 (12)	C24—C23—H23	119.9
O21—C121—H121	108.4	C22—C23—H23	119.9
N1—C121—H121	108.4	C23—C24—C25	120.62 (14)
C12—C121—H121	108.4	C23—C24—H24	119.7
C14—C13—C12	120.10 (14)	C25—C24—H24	119.7
C14—C13—H13	120	C26—C25—C24	119.59 (14)
C12—C13—H13	120	C26—C25—H25	120.2
C13—C14—C15	120.80 (14)	C24—C25—H25	120.2
C13—C14—H14	119.6	O26—C26—C25	125.72 (14)
C15—C14—H14	119.6	O26—C26—C21	114.69 (13)
C16—C15—C14	119.99 (14)	C25—C26—C21	119.56 (14)
C16—C15—H15	120	C26—O26—C261	116.82 (12)
C14—C15—H15	120	O26—C261—H26A	109.5
O16—C16—C15	125.65 (14)	O26—C261—H26B	109.5
O16—C16—C11	115.10 (13)	H26A—C261—H26B	109.5
C15—C16—C11	119.24 (14)	O26—C261—H26C	109.5
C16—O16—C161	116.22 (11)	H26A—C261—H26C	109.5
O16—C161—H16A	109.5	H26B—C261—H26C	109.5
O16—C161—H16B	109.5		

### Hydrogen-bond geometry (Å, º)

**Table:**

*D*—H···*A*	*D*—H	H···*A*	*D*···*A*	*D*—H···*A*
C13—H13···O11^i^	0.95	2.69	3.5616 (19)	152
C1—H1*B*···O26^ii^	0.98	2.53	3.508 (2)	176

Symmetry codes: (i) *x*−1, *y*, *z*; (ii) −*x*+1, −*y*+1, −*z*+2.
