# Supplementary material for: Crystal structure of 4,10-dimeth­oxy-13-methyl-6H,12H-6,12-epimino­dibenzo[b,f][1,5]dioxocine
Source: Acta Crystallogr E Crystallogr Commun. 2017 Feb 21;73(Pt 3):410–2. doi: 10.1107/S2056989017002328 (PMC5347066; doi:10.1107/S2056989017002328)
Supplement: Supplementary file 3 [file e-73-00410-sup3.pdf]

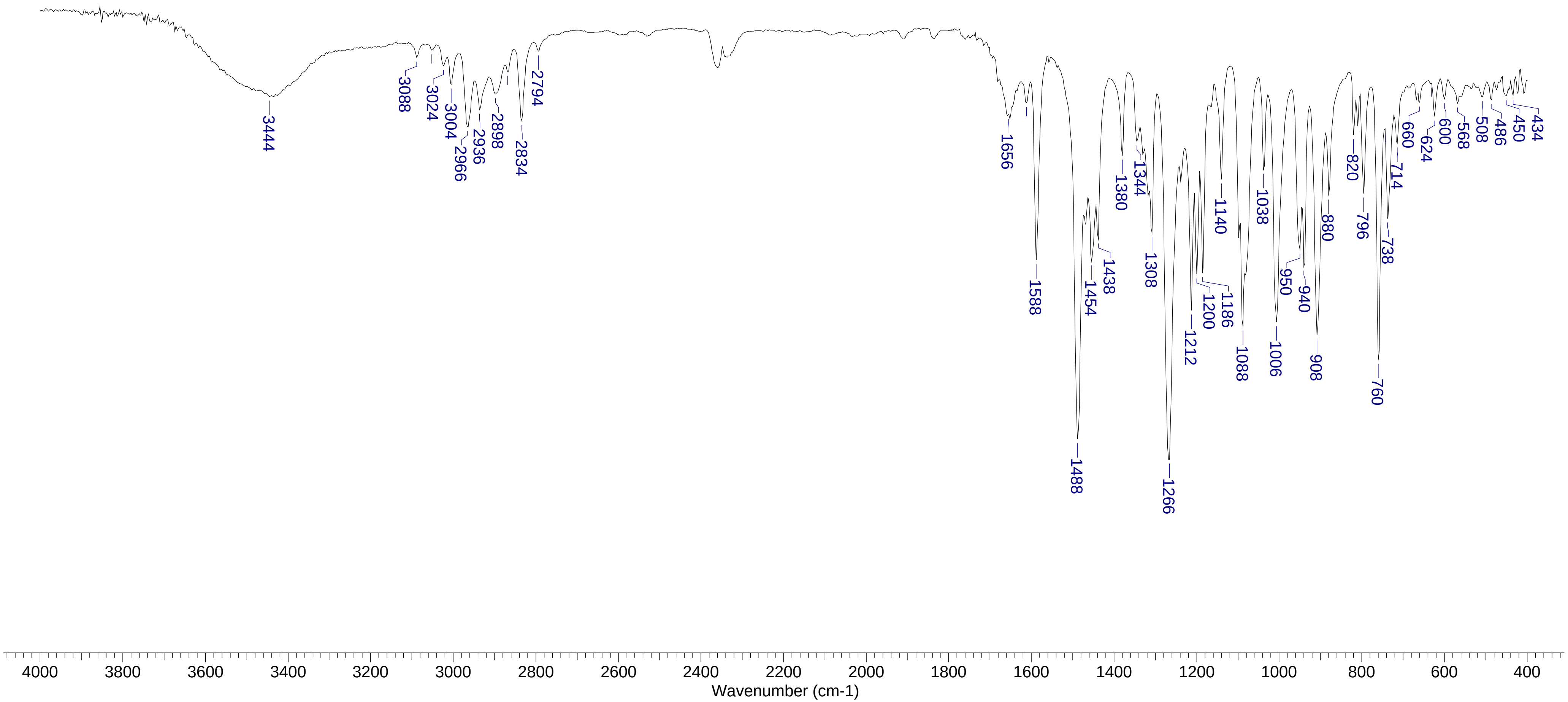

No	cm-1	%T	Intensity
1	434.00	85.308	W
2	450.00	85.181	W
3	486.00	84.609	W
4	508.00	85.091	W
5	568.00	84.039	W
6	600.00	84.772	W
7	624.00	81.951	W
8	632.00	87.223	W
9	660.00	84.245	W
10	714.00	77.751	M
11	738.00	65.933	M
12	744.00	80.097	W
13	760.00	43.634	S
14	796.00	69.848	M
15	820.00	79.086	W
16	880.00	69.579	M
17	908.00	47.536	S
18	940.00	58.322	M
19	950.00	61.044	M
20	1006.00	49.569	S
21	1038.00	73.630	M
22	1088.00	48.893	S
23	1140.00	72.114	M
24	1186.00	57.323	M
25	1200.00	57.088	M
26	1212.00	51.411	S
27	1266.00	27.948	VS
28	1308.00	63.653	M
29	1344.00	78.087	W
30	1380.00	75.836	M
31	1438.00	62.662	M
32	1454.00	59.208	M
33	1488.00	31.134	VS
34	1588.00	59.364	M
35	1612.00	84.128	W
36	1656.00	82.274	W
37	2794.00	92.286	VW
38	2834.00	81.300	W
39	2868.00	89.037	W
40	2898.00	85.542	W
41	2936.00	83.091	W
42	2966.00	80.360	W
43	3004.00	87.066	W
44	3024.00	89.996	W
45	3052.00	92.462	VW
46	3088.00	91.310	W
47	3444.00	85.125	W
